# Integral-Valued Pythagorean Fuzzy-Set-Based Dyna Q+ Framework for Task Scheduling in Cloud Computing

**DOI:** 10.3390/s24165272

**Published:** 2024-08-14

**Authors:** Bhargavi Krishnamurthy, Sajjan G. Shiva

**Affiliations:** 1 Department of Computer Science and Engineering, Siddaganga Institute of Technology, Tumakuru 572103, Karnataka, India; 2 Department of Computer Science, University of Memphis, Memphis, TN 38152-3240, USA

**Keywords:** Dyna-Q-learning, cloud computing, interval-valued Pythagorean fuzzy set, uncertainty, performance

## Abstract

Task scheduling is a critical challenge in cloud computing systems, greatly impacting their performance. Task scheduling is a nondeterministic polynomial time hard (NP-Hard) problem that complicates the search for nearly optimal solutions. Five major uncertainty parameters, i.e., security, traffic, workload, availability, and price, influence task scheduling decisions. The primary rationale for selecting these uncertainty parameters lies in the challenge of accurately measuring their values, as empirical estimations often diverge from the actual values. The integral-valued Pythagorean fuzzy set (IVPFS) is a promising mathematical framework to deal with parametric uncertainties. The Dyna Q+ algorithm is the updated form of the Dyna Q agent designed specifically for dynamic computing environments by providing bonus rewards to non-exploited states. In this paper, the Dyna Q+ agent is enriched with the IVPFS mathematical framework to make intelligent task scheduling decisions. The performance of the proposed IVPFS Dyna Q+ task scheduler is tested using the CloudSim 3.3 simulator. The execution time is reduced by 90%, the makespan time is also reduced by 90%, the operation cost is below 50%, and the resource utilization rate is improved by 95%, all of these parameters meeting the desired standards or expectations. The results are also further validated using an expected value analysis methodology that confirms the good performance of the task scheduler. A better balance between exploration and exploitation through rigorous action-based learning is achieved by the Dyna Q+ agent.

## 1. Introduction

Today’s business environment is very complex and cannot easily be supported by traditional IT solutions due to the explosive growth in application sizes, large volume of e-content generation, exponential growth in the computing capability of devices, introduction of newer architectures, etc. Cloud computing is one of the extremely important on-demand computing platforms used to perform tasks instead of these being performed using self-configurable computing resources. Several distinct properties of cloud computing include multitenancy, elasticity, pay per use features, resiliency, workload movement ease, and so on. Several advantages offered by high-end computation include scalability, high performance, intense computational power, better availability, reduced cost of operation, and many more [1,2,3].

Despite all the advantages, cloud computing is subject to a lot of challenges pertaining to security, cost of operation, resource management, multi-cloud management, performance, segmented adaption, application migration, interoperability, reliability, and availability. Out of all the challenges, performance-related challenges (task scheduling, load balancing, and resource management) are of paramount importance because good performance is vital for the overall success of cloud computing. Poor performance leads to the dissatisfaction of users and, in turn, a decrease in revenue generation. Also, it introduces hindrances to the seamless successful execution of high-end applications [4,5,6].

Task scheduling, being a paramount performance concern, has garnered significant attention from researchers over the past few decades. Precise scheduling of tasks is considered a nondeterministic polynomial time hard (NP-Hard) problem because it is difficult to find near-optimal solutions within the stipulated time limits under conditions of uncertainty using classical algorithms. Machine learning algorithms have been found to be very promising in tackling task scheduling problems [7]. However, these algorithms also suffer from lower convergence rates, and higher tendencies to converge toward a local optimal solution. Hence, there is a need to develop intelligent, uncertainty-proof algorithms by properly balancing exploration and exploitation activities and to achieve enhanced results in very few iterations of training [8]. The existing task scheduling algorithms have limitations in managing uncertainty, leading to higher task failure rates. They are often reactive, stochastic, and fuzzy, lacking adaptability and dynamic computing capabilities, and tend to converge on suboptimal solutions.

Uncertainty is one of the main issues that affect the computing efficiency of cloud computing. Five major uncertainty parameters in cloud computing are security, traffic, availability, price, and workload. Vital sources of uncertainty are data (variety, value), virtualization, job arrival rates, job migration rates, energy consumption, fault tolerance, scalability, dynamic pricing, resource availability, elasticity, consolidation, communication, replication, elastic provisioning, etc. The performance metrics affected due to uncertainty are throughput, scalability, cost, adaptability, accuracy, transparency, and response time. Hence, there is a necessity to efficiently handle the parameters causing uncertainty and then make intelligent task scheduling decisions [9,10].

The integral-valued Pythagorean fuzzy set (IVPFS) is a modification of the fuzzy set. The IVPFS is based on intuitionistic fuzzy sets. The uncertainty is represented as the membership and non-membership degree in terms of the integral value, which is in the range of [0, 1]. With respect to the IVPFS, the sum of the square of membership and non-membership values is less than 1. The IVPFS, composed of operators like concentration, dilation, and normalization, helps in handling imprecise, incomplete, and inadequate data to express an opinion in precise numerical values [11,12].

The Dyna Q+ algorithm is a modified form of the Dyna Q algorithm [13]. The Dyna Q+ learning agent receives bonus rewards for actions that have not been carried out for a longer time. It updates the agent’s rewards based on time. If the Q-learning agent has visited a state long back, the rewards obtained will be increased, which will empower the agent to visit that particular state again. The Dyna Q+ agent is suitable for dynamically changing environments and provides exploration bonus rewards that encourage exploration activity [14]. Some potential applications of the proposed work include resource allocation, cost management, load balancing, production planning, inventory management, and maintenance scheduling.

In this paper, the Dyna Q+ algorithm is made uncertainty-proof with the application of the IVPFS mathematical framework. The IVPFS mathematical model exhibits excellent ability to handle imprecise and vague parameters of tasks and virtual machines. The Dyna Q+ learning agent is designed to adapt to the changing dynamics of cloud systems. Scheduling policies are formulated through vigorous action-based learning.

The main objectives of the paper are as follows:

- A mathematical representation of the cloud computing system model is constructed for task scheduling, and definitions of the performance metrics are given;
- Mathematical definitions of the performance metrics are set to evaluate the efficiency of the proposed framework;
- A novel IVPFS-Dyna Q+ task scheduler is designed with the supporting algorithms as a component of the framework;
- The IVPFS-Dyna Q+ task scheduler is simulated using the CloudSim 3.3 simulator by considering three different types of workloads: a random dataset, GOCJ dataset, and synthetic dataset;
- The results are validated through expected value analysis of the proposed IVPFS-Dyna Q+ task scheduler.

The remaining part of the paper is organized as follows: Section 2 discusses the existing works. Section 3 presents the cloud system model considered for operation along with the definitions of the performance metrics. Section 4 presents the proposed IVPFS-enabled task scheduler with two subcomponents: the interval-valued Pythagorean fuzzy set resource pool (IVPFS_RP), and the interval-valued Pythagorean fuzzy set client workflow (IVPFS_CWF). Section 5 presents an expected value analysis of the proposed work. Section 6 presents the results and discussion, and finally, Section 7 presents the conclusion.

## 2. Related Work

Tong et al. [15] present a novel task scheduling scheme based on Q-learning embedded with a heterogeneous earliest finish time policy. The scheme works in two phases. The first phase consists of sorting the available list of tasks in the optimal order using Q-learning. The second phase involves allocating the processor for the tasks using the earliest finish time policy. The static scheduling problem is solved using the proposed scheduling scheme. By providing immediate rewards, the Q-learning agent is made to go through a better learning experience. The immediate reward for each action is provided using the upward rank. After every action, the Q-table is updated through a self-learning process. The performance is tested against several benchmarks, and the results obtained reveal a significant reduction in makespan time and response time. However, the scheme leads to an overestimation of policies and may be too optimistic in policy formation.

Kruekaew et al. [16] discuss a hybrid bee colony algorithm with a reinforcement learning technique embedded to balance the load of virtual machines in the cloud. The main goal of load balancing is to ensure that the load is balanced across all virtual machines. It must be ensured that none of the virtual machines are overloaded or underloaded. By applying reinforcement for every action by the agent, the speed of the bee colony algorithm is enhanced. Task scheduling decisions are made by making predictions using an appropriate scheduling table. A mathematical model is formulated to include the following performance metrics: cost of operation, resource utilization, and makespan time. The algorithm is tested on the CloudSim simulator by considering three random datasets, and the performance is good with respect to resource utilization and throughput. However, the performance of the scheduler is not optimized on every dataset considered for evaluation. There might be a chance that it generates poor-quality solutions and that it converges toward suboptimal solutions.

Hou et al. [17] present a specialized review of the energy efficient task scheduling algorithm based on deep reinforcement learning for cloud computing. Energy consumption is of primary concern in cloud data centers. The potential of deep reinforcement learning (DRL) is to make energy efficient task scheduling decisions. First, a classification of energy models in the cloud data centers is carried out. An energy consumption model is developed by considering the energy consumed by the data centers. However, measuring the power consumed in every partition is challenging in practical scenarios. The existing DRL methods are analyzed by considering several benchmarks with respect to type, space, state, action, and reward metrics. A brief guideline is provided for the formulation of a reward function and the objective to be considered while scheduling tasks. From the survey, it was found that there is a lack of performance comparisons between DRL scheduling algorithms. Even the effectiveness of the algorithms is not determined with respect to policy formulation and value computation.

Neelakantan et al. [18] discuss an optimized machine learning strategy to effectively schedule the jobs in the cloud. Job scheduling for cloud environments is considered a problematic job as these environments have heterogeneous operating systems and necessitate user requirement validation by a virtual machine before scheduling. A novel hybrid framework composed of a convolutional neural network and whale optimization strategy (CNN-W) was proposed for task scheduling. The cloud framework is composed of a fixed number of virtual machines for task execution. The deadline of the tasks was considered a metric for performing scheduling using CNN-W. In order to reduce resource consumption and the task execution time, the deadline was given higher priority. The framework first allocated the tasks, followed by deadline prediction and priority setting. Priorities were assigned based on the duration of tasks. Short-duration tasks were given higher priority, and the remaining jobs were given lower priority. The performance of the framework was tested on the Python platform by considering several benchmark datasets. The prediction accuracy was enhanced, but the fault tolerance was not considered. As a result, the virtual machines became more vulnerable to damage. The robustness score was lower, and because of this, the task execution performance was low.

Attiya et al. [19] discussed a hybrid algorithm combining the Manta ray foraging optimization (MRFO) and salp warm optimization (SSO) algorithms for the scheduling of internet of things (IoT) tasks in the cloud. The MRFO metaheuristic algorithm uses three types of foraging operators, i.e., chain, cyclone, and somersault, for solving the optimization problem. The SSO is another metaheuristic algorithm inspired by the swarming behavior of salps in the ocean. The random selection of reference points in MRFO leads to weakened search capability for promising solutions. However, the search ability of MRFO is improved by incorporating SSO. The performance of MRFO-SSA was tested by considering different real-world datasets, which resulted in higher throughput and an improved convergence rate. However, the MRFO-SSA was not able to balance between exploration and exploitation operations.

The drawbacks observed in the existing works are as follows:

- Uncertainties of the task and resource parameters are poorly or insufficiently modeled;
- They show an inability to search within the large search spaces of cloud systems, resulting in a low probability of arriving at a global optimal solution;
- They come with a high probability of task failure due to the improper mapping of tasks to resources;
- Fundamental approaches available in the literature are reactive, stochastic, and fuzzy, and these approaches lack adaptability, dynamic computing ability, and the ability to converge towards suboptimal solutions;
- The robustness scores achieved are lower as cloud resources are more vulnerable to becoming damaged;
- There is an improper balance between exploration and exploitation, which results in poor task scheduling policies;
- Existing task scheduling policies are static as they do not deal with highly dynamic cloud scenarios;
- Some of the task schedulers are inflexible in handling multi-cloud environments as they are trained for specific types of cloud environment;
- Scheduling policies are found to violate SLA due to ineffective scheduling.

## 3. System Model

Consider a typical cloud computing environment involving *m* and the collection of resource pools, RP = $<{RP}_{1},{RP}_{2},{RP}_{3},\ldots ,{RP}_{m}>$, where *m* represents the total count of virtual machines. Each of the resources in ${RP}_{i}$ has resources like RAM, CPU, and bandwidth. Similarly, *n* and the independent collection of client workflows are available for execution, ${WF}_{i}=<{WF}_{1},{WF}_{2},{WF}_{3},\ldots ,{WF}_{n}>$, where *n* represents the total count of tasks. The uncertainty in the resource and client workflow are handled by applying the IVPFS, i.e., $IVPFS\_RP=IVPFS\_RP<IVPFS\_{RP}_{1},\ldots ,{IVPFS\_RP}_{m}>$, and $IVPFS\_WF=<IVPFS\_{WF}_{1},\ldots ,{IVPFS\_WF}_{n}>$.

The system model determines the stability of an environment for task scheduling by considering sensitive performance objectives like workflow execution time, WFET(Dyna Q+); makespan time, $MST\left(Dyna\;Q+\right)$; operation cost, OC(Dyna Q+); and resource utilization rate, RU(Dyna Q+). An optimal solution is designed by computing the fitness of the scheduling solution. The main performance objectives of the proposed framework are defined below:

**PO1: Workflow Execution Time** (WFET(Dyna Q+)): This is the time taken by the cloud system to complete the last workflow.

(1) $$WFET(Dyna\;Q+({RP}_{i}))=\sum_{T_{i}\in {vm}_{i}}\frac{\sum length({WF}_{i})}{CPU({RP}_{i})}$$

where the $length({WF}_{i})$ is defined as the summation of the number of instructions taken by each of the workflows in the workflow, set ${WF}_{i}\subseteq WF$, to be executed, and $CPU({RP}_{i})$ is the CPU rate of ${RP}_{i}$ for processing.

The fitness of $WFET(Dyna\;Q+({RP}_{i}))$ is determined as the ratio of the minimum workflow execution time to the actual workflow execution time.

(2) $$F(WFET(Dyna\;Q+({RP}_{i})))=\frac{Min(WFET(Dyna\;Q+({vm}_{i}))}{WFET(Dyna\;Q+({vm}_{i}))}$$

**PO2: Makespan Time** (MST(Dyna Q+)): This is defined as the maximum workflow execution time of all the resource pools in the cloud system.

(3) $$MST\left(Dyna\;Q+({RP}_{i})\right)=Max(WFET(Dyna\;Q+({RP}_{i})))$$

The fitness of $MST(Dyna\;Q+({RP}_{i}))$ is determined as the ratio of the minimum makespan time to the actual makespan time.

(4) $$F(MST(Dyna\;Q+({RP}_{i})))=\frac{Min(MST(Dyna\;Q+({RP}_{i}))}{MST(Dyna\;Q+({RP}_{i}))}$$

**PO3: Operation Cost** (OC(Dyna Q+)): This is defined as the cost incurred by the resource pool in processing the requests.

(5) $$OC\left(Dyna\;Q+\right)=\sum_{i=1}^{m}\left(C_{1}\;*\;TET(Dyna\;Q+({RP}_{i})))+(C_{2}\;*\;TET(Dyna\;Q+({RP}_{i})))+(C_{3}\;*\;TET(Dyna\;Q+({RP}_{i})))\right)$$

where $C_{1}$ represents the CPU usage cost, $C_{2}$ represents the memory usage cost, and $C_{3}$ represents the bandwidth usage cost.

The fitness of *F*(OC (Dyna Q+)) is determined as the ratio of the minimum operation cost to the operation cost.

(6) $$F(OC(Dyna\;Q+({RP}_{i})))=\frac{Min(OC(Dyna\;Q+({RP}_{i}))}{OC(Dyna\;Q+({RP}_{i}))}$$

**PO4: Resource utilization rate** (RU(Dyna Q+)): This is defined as the summation of the memory load on the resource pool, $LM\left({vm}_{i}\right)$, and the CPU load on the resource pool, $LC\left({RP}_{i}\right)$.

(7) $$RU\left(Dyna\;Q+\right)=\sum_{i=1}^{i=m}\left(LM\left({RP}_{i}\right)+LC\left({RP}_{i}\right)\right)$$

where $LM\left({RP}_{i}\right)$ is computed by considering the memory used before the execution of the task set, $BM\left(T_{i}\right)$, the memory occupied by the workflow, ${WF}_{i}OM\left({WF}_{i}\right)$, and the total memory available in the resource pool, $TM\left({RP}_{i}\right)$.

(8) $$LM\left({RP}_{i}\right)=BM\left({WF}_{i}\right)+\frac{OM\left({WF}_{i}\right)}{TM(\left({RP}_{i}\right)}$$

Similarly $LC\left({RP}_{i}\right)$ is computed by considering the CPU used before the execution of the workflow, $BC\left({WF}_{i}\right)$, the CPU occupied by the workflow, $OM\left({WF}_{i}\right)$, and the total CPU available in the resource pool, $TM(\left({RP}_{i}\right)$.

(9) $$LC\left({RP}_{i}\right)=BC\left({WF}_{i}\right)+\frac{OC\left({WF}_{i}\right)}{TC(\left({RP}_{i}\right)},$$

The fitness of *F(RU*(Dyna Q+)) is determined as the ratio of the weighted proportion of $LM\left({RP}_{i}\right)$ and $LM\left({RP}_{i}\right)$.

(10) $$F(RU(Dyna\;Q+))=\frac{w_{1}}{1-LM\left({RP}_{i}\right)}\;*\;\frac{w_{2}}{1-LC\left({RP}_{i}\right)}$$

where $w_{1}$ and $w_{2}$ are the weights assigned to the memory and CPU, such that $w_{1}+w_{2}=1$.

The overall fitness function is computed as follows:

(11) $$\begin{array}{ll} F\left(Dyna\;Q+\right)= & \gamma_{1}*F\left(TET\left(Dyna\;Q+\left({RP}_{i}\right)\right)\right)+\gamma_{2}*F\left(MST\left(Dyna\;Q+\left({RP}_{i}\right)\right)\right)+\gamma_{3}\\ & *F\left(OC\left(Dyna\;Q+\left({RP}_{i}\right)\right)\right)+\gamma_{4}*F\left(RU\left(Dyna\;Q+\left({RP}_{i}\right)\right)\right) \end{array}$$

where, $\gamma_{1}$, $\gamma_{2}$, and $\gamma_{3}$ are the balance coefficients required to determine optimal solution. A higher fitness function value leads to an optimal solution.

## 4. Proposed Work

The proposed work is mainly composed of three subcomponents, which are the interval-valued Pythagorean fuzzy set resource pool (IVPFS_RP), interval-valued Pythagorean fuzzy set client workflow (IVPFS_WF), and Dyna Q+ task scheduler. The IVPFS_RP is responsible for removing the parametric uncertainties in the virtual machine resource pool. Similarly, the IVPFS_WF is responsible for reducing the parametric uncertainties in the client workflow. The Dyna Q+ task scheduler generates the scheduling policies over the reduced set of resources pool and client workflows. The Dyna Q+ agent mainly executes Dyna Q logic and provides additional bonus rewards for actions that are left pending for a longer duration through the exploration activity.

### 4.1. IVPFS_RP

The virtual machine resources in cloud computing systems are associated with several forms of uncertainties, which include network congestion, improper placement of resources, loss of data, inadequate processor cores, compatibility problems, frequent repartitioning, more downtime, resource contention between the collocated virtual machines, overloading of resources, random variation in the processing capability, and so on. The uncertainties in the resources are reduced with the application of the IVPFS.

The IVPFS form of the resource pool, ${RP}_{i}$, is defined as follows.

$IVPFS\left({RP}_{i}\right)=\{<{RP}_{i},\left[\mu^{l}\left({RP}_{i}\right),\mu^{u}\left({RP}_{i}\right)\right],\left[V^{l}\left({RP}_{i}\right),V^{u}\left({RP}_{i}\right)\right]>:{RP}_{i}\in RP\}$, where $\mu^{l}\left({RP}_{i}\right),\mu^{u}\left({RP}_{i}\right)$ represent the lower and upper membership degrees of ${RP}_{i}$, and $V^{l}\left({RP}_{i}\right),V^{u}\left({RP}_{i}\right)$ represent the lower and upper non-membership degrees of ${WF}_{i}$. These satisfy the conditions $1\geq \mu^{u}\left({RP}_{i}\right)\geq \mu^{l}\left({RP}_{i}\right)\geq 0$ and $0\leq V^{u}\left({RP}_{i}\right)\leq V^{l}\left({RP}_{i}\right)\leq 1$. An additional constraint is $0\leq {\mu^{l}\left({RP}_{i}\right)}^{2}+{V^{l}\left({RP}_{i}\right)}^{2}\leq 1$, and $0\leq {\mu^{u}\left({RP}_{i}\right)}^{2}+{V^{u}\left({RP}_{i}\right)}^{2}\leq 1$. The approximate degree of $IVPFS\left({RP}_{i}\right)$ is computed as follows:

(12) $$\prod {RP}_{i}=[\pi^{l}\left({RP}_{i}\right),\;\pi^{u}\left({RP}_{i}\right)]$$

(13) $$\prod {RP}_{i}=[\sqrt{1-{\mu^{l}\left({RP}_{i}\right)}^{2}-{V^{l}\left({RP}_{i}\right)}^{2}}\;,\;\sqrt{1-{\mu^{u}\left({RP}_{i}\right)}^{2}-{V^{u}\left({RP}_{i}\right)}^{2}}]$$

The workflow of the IVPFS_RP is provided in Algorithm 1.
**Algorithm 1:** Working of IVPFS_RP1: **Begin** 2: Input: $RP=<{RP}_{1},{RP}_{2},{RP}_{3},\ldots ,{RP}_{n}>$ 3: Output:$\;IVPFS\_RP=<IVFFN\_{RP}_{1},\ldots ,{IVFFN\_RP}_{m}>$ 4: **Training phase of IVPFS_RPUR** 5: **for each** incoming training client workflow ${RP}_{i}\in RP$
**do** 6:    Compute lower and upper membership degree of ${RP}_{i}$   $\mu^{l}\left({RP}_{i}\right),\;\mu^{u}\left({RP}_{i}\right)$ 7:    Compute lower and upper non membership degree of ${WF}_{i}$                $V^{l}\left({RP}_{i}\right),\;V^{u}\left({RP}_{i}\right)$ 8:  **end for** 9: **Testing: IVPFS_RPUR** 10: **for** every incoming test client workflow ${RP}_{i}\in RP$
**do** 11:    Compute lower and upper membership degree of ${RP}_{i}$   $\mu^{l}\left({RP}_{i}\right),\;\mu^{u}\left({RP}_{i}\right)$ 12:    Compute lower and upper non membership degree of ${RP}_{i}$                $V^{l}\left({RP}_{i}\right),\;V^{u}\left({RP}_{i}\right)$ 13:  **end for** 14:   Enumerate IVPFS_RP       $IVPFS\_RP∷=IVPFS\_{RP}_{i}\cup (\mu^{l}\left({RP}_{i}\right),\;\mu^{u}\left({RP}_{i}\right),V^{l}\left({RP}_{i}\right),\;V^{u}\left({RP}_{i}\right))$ 15: **Output** IVFTFS_RP resource pool          $IVPFS\_RP=<IVPFS\_{RP}_{1},\ldots ,{IVPFS\_RP}_{n}>$ 16: **End**

Client workflows in cloud computing systems are associated with several forms of uncertainties, which include variations in the task arrival rate, poor data representation, fluctuations in the data volume, frequent pre-emption of tasks, unrealistic task deadlines, improper task deployment, task parallelization, failure of task execution, high energy consumption, and so on.

The IVPFS form of the client workflow, ${WF}_{i}$, is defined as follows.

$IVPFS\left({WF}_{i}\right)=\{<{WF}_{i},\left[\mu^{l}\left({WF}_{i}\right),\mu^{u}\left({WF}_{i}\right)\right],\left[V^{l}\left({WF}_{i}\right),V^{u}\left({WF}_{i}\right)\right]>:{WF}_{i}\in WF\}$, where $\mu^{l}\left({WF}_{i}\right),\mu^{u}\left({WF}_{i}\right)$ represent the lower and upper membership degrees of ${WF}_{i}$, and $V^{l}\left({WF}_{i}\right),{\;V}^{u}\left({WF}_{i}\right)$ represent the lower and upper non-membership degrees of ${WF}_{i}$. These satisfy the condition $1\geq \mu^{u}\left({WF}_{i}\right)\geq \mu^{l}\left({WF}_{i}\right)\geq 0$ and $0\leq V^{u}\left({WF}_{i}\right)\leq V^{l}\left({WF}_{i}\right)\leq 1$. Additional constraints include $0\leq {\mu^{l}\left({WF}_{i}\right)}^{2}+{V^{l}\left({WF}_{i}\right)}^{2}\leq 1$ and $0\leq {\mu^{u}\left({WF}_{i}\right)}^{2}+{V^{u}\left({WF}_{i}\right)}^{2}\leq 1$. The approximate degree of $IVPFS\left({WF}_{i}\right)$ is computed as follows:

(14) $$\prod {WF}_{i}=[\pi^{l}\left({WF}_{i}\right),\;\pi^{u}\left({WF}_{i}\right)]$$

(15) $$\prod {WF}_{i}=[\sqrt{1-{\mu^{l}\left({WF}_{i}\right)}^{2}-{V^{l}\left({WF}_{i}\right)}^{2}}\;,\;\sqrt{1-{\mu^{u}\left({WF}_{i}\right)}^{2}-{V^{u}\left({WF}_{i}\right)}^{2}}]$$

The protocol of the IVPFS_WF is provided in Algorithm 2.
**Algorithm 2:** Working of IVPFS_WFUR1: **Begin** 2: Input: $WF=<{WF}_{1},{WF}_{2},{WF}_{3},\ldots ,{WF}_{m}>$ 3: Output:$\;IVPFS\_WF=<IVPFS\_{WF}_{1},\ldots ,{IVPFS\_WF}_{M}>$ 4: **Training phase of IVPFS_WF** 5: **for each** incoming training client workflow ${WF}_{i}\in WF$
**do** 6:    Compute lower and upper membership degree of ${WF}_{i}$                $\mu^{l}\left({WF}_{i}\right),\;\mu^{u}\left({WF}_{i}\right)$ 7:    Compute lower and upper non membership degree of ${WF}_{i}$                $V^{l}\left({WF}_{i}\right),\;V^{u}\left({WF}_{i}\right)$ 8:   **end for** 9: **Testing: IVPFS_WF** 10: **for** every incoming test client workflow ${WF}_{i}\in WF$
**do** 11:    Compute lower and upper membership degree of ${WF}_{i}$                $\mu^{l}\left({WF}_{i}\right),\;\mu^{u}\left({WF}_{i}\right)$ 12:    Compute lower and upper non membership degree of ${WF}_{i}$                $V^{l}\left({WF}_{i}\right),\;V^{u}\left({WF}_{i}\right)$ 13:  **end for** 14:   Enumerate IVPFS_WF       $IVPFS\_WF∷=IVPFS\_{WF}_{i}\cup (\mu^{l}\left({WF}_{i}\right),\;\mu^{u}\left({WF}_{i}\right),V^{l}\left({WF}_{i}\right),\;V^{u}\left({WF}_{i}\right))$ 15: **Output** IVPFS_WF client workflows          $IVPFS\_WF=<IVPFS\_{WF}_{1},\ldots ,{IVPFS\_WF}_{m}>$ 16: **End**

### 4.2. Proposed IVPFS Based Dyna Q+ Task Scheduler

**Figure 1:**
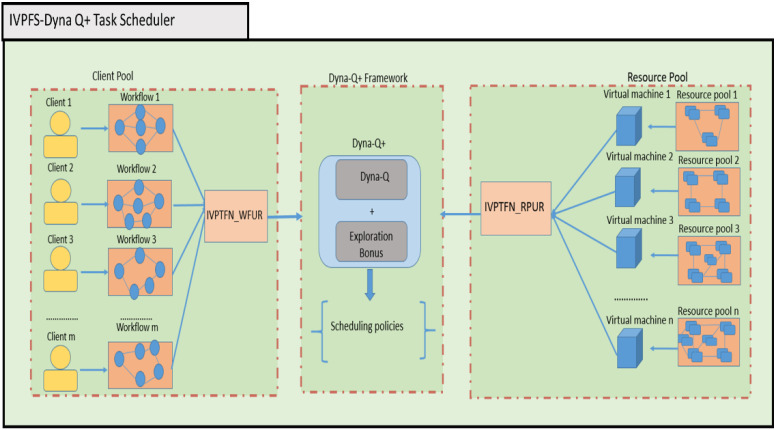
Proposed IVPFS-Dyna Q+ task scheduler.

The high-level architecture of the proposed IVPFS Dyna Q+ task scheduler is shown in Figure 1. It is composed of three main components: the client pool, Dyna Q+ framework, and resource pool. In the client pool component, the incoming clients submit their workflows, and each client is composed of varying degrees of workflows. The uncertainties involved in the client workflow are reduced by the functional module IVPFS_WF. Similarly, the resource pool component is composed of virtual machines with varying degree of resources. The uncertainties involved in the virtual machine resource pool are reduced using the IVPFS_RP. Finally, the Dyna Q+ framework is responsible for formulating the task scheduling policies. The Dyna Q+ framework mainly executes Dyna Q logic and provides an additional bonus reward for the actions that are left pending for a longer duration through the exploration activity. The workflow of the proposed IVPFS-based Dyna Q+ task scheduler is provided in Algorithm 3.
**Algorithm 3:** Working of IVPFS-Dyna Q+ task scheduler1: **Begin** 2: **Input:**          $IVPFS\_WF=<IVPFS\_{WF}_{1},\ldots ,{IVPFS\_WF}_{m}>$           $IVPFS\_RP=<IVPFS\_{RP}_{1},\ldots ,{IVPFS\_RP}_{n}>$ 3: **Output:**          $\prod \left(IVPFS\_WF\to IVPFS\_RP\right)=<\pi_{i},\ldots .,\pi_{k}>$
4: **Training Phase: IVPFS-Dyna Q+ task scheduler** 5: Initialize Q(S,A)=⊘ and Model(S,A)=⊘ for all s∈Sanda∈A 6: **for** every training $IVPFS\_{WF}_{i}$ and $IVPFS\_{RP}_{j}\;$**do** 7:  **Begin the Dyna Q+ model learning phase** 8:    Initialise the Q+ learning agent state St← current non-terminal state 9:    Perform the action Aa ←ϵgreedy policy (St, Aa) 10:     Take an action Aa, in which ${Aa}_{i}\in Aa$, change the state from $S\;to\;S^{1}$ 11:     Calculate the Q value       $Q\left(St,Aa\right)=Q\left(St,Aa\right)+\alpha [Rt+\gamma {max}_{a}Q\left({St}^{1},Aa\right)-Q\left(St,Aa\right)]$ 12:   **End the Dyna Q+ model learning phase** 13:   **Begin the Dyna Q+ real interaction learning phase** 14:       Get a robot to some random state St← current non-terminal state 15:       Generate an action Aa ← Robot Experience (St, Aa) 16:         Execute the action Aa in the environment 17:         Update the model, save the action ${Rt}_{i}\in Rt$, and move to next state ${St}^{1}$ 18:           Update the Q value: Send to the Dyna Q+ model learning phase           $UQ\left(St,Aa\right)=UQ\left(St,Aa\right)+\alpha [Rt+\gamma {max}_{a}Q\left({St}^{1},Aa\right)-Q\left(St,Aa\right)]$ 19:   **End the Dyna Q+ real interaction learning phase** 20: **End for** 21: Formulate policies $\Pi ∷=\Pi ⋃\Pi_{i}$ 22: **Testing Phase: IVPFS-Dyna Q+ task scheduler** 23: Initialize Q(S,A)=⊘ and Model(S,A)=⊘ for all s∈Sanda∈A 24: every testing $T2SS\_{CT}_{i}$ and ${T2SS\_GR}_{i}$ **do** 25: **Begin the Dyna Q+ model testing phase** 26:      Take an action Aa, where Aa∈A, update the state $St\to {St}^{1}$ 27:        Compute the Q value       $Q\left(St,Aa\right)=Q\left(St,Aa\right)+\alpha [Rt+\gamma {max}_{a}Q\left({St}^{1},Aa\right)-Q\left(St,Aa\right)]$ 28: **End the Dyna Q+ model testing phase** 29: **Begin the Dyna Q+ real interaction testing phase** 30:     Update the testing model $Model\left(S,A\right)\leftarrow R$ and $Model\left(S,A\right)\leftarrow S^{1}$ 31:     Update the Q value: Send to the Dyna Q+ model testing phase      $UQ\left(St,Aa\right)=UQ\left(St,Aa\right)+\alpha [Rt+\gamma {max}_{a}Q\left({St}^{1},Aa\right)-Q\left(St,Aa\right)]$ 32: **End the Dyna Q+ real interaction testing phase** 33: Output the $\prod \left(IVPFS\_WF\to IVPFS\_RP\right)=$ $<\Pi_{1},\Pi_{2},\ldots ,\Pi_{p}>$
34: **End**

## 5. Expected Value Analysis

The expected value analysis of the proposed Dyna Q+ task scheduler is performed by considering three of the recent existing works (E1, E2, and E3). The four performance metrics (POs) considered for analysis are the workflow execution time, makespan time, operation cost, and resource utilization rate.

**PO1: Workflow Execution Time** (WFET(Dyna Q+)): The expected task execution time ℮(WFET(Dyna Q+)) is influenced by two factors, the expected length of the workflow, $℮(length\left({WF}_{i}\right))$, and the expected CPU utilization rate of the resource pool, $℮(CPU\left({RP}_{i}\right))$.

$$℮\left(\frac{WFET\left(Dyna\;Q+\right)}{℮\left(length\left({WF}_{i}\right)\right),℮(CPU\left({RP}_{i}\right))\;},\;T\right)=\int_{\alpha }^{\infty }\frac{\sum_{\alpha \epsilon \phi }℮(WFET(Dyna\;Q+))}{\left|℮\left(length\left({WF}_{i}\right)\right),℮(CPU\left({RP}_{i}\right))\right|}$$

$$℮\left(\frac{WFET\left(Dyna\;Q+\right)}{℮\left(length\left({WF}_{i}\right)\right),℮(CPU\left({RP}_{i}\right))\;},\;T\right)=\sum_{c\in C}c\int_{\alpha }^{\infty }\frac{\sum_{\alpha \epsilon \phi }℮\left(WFET\left(Dyna\;Q+\right)\right)(a)}{\left|℮\left(length\left({WF}_{i}\right)\right),℮(CPU\left({RP}_{i}\right))\right|}$$

$$℮\left(\frac{WFET\left(Dyna\;Q+\right)}{℮\left(length\left({WF}_{i}\right)\right),℮(CPU\left({RP}_{i}\right))\;},\;T\right)=℮\left(\frac{1*℮\left(length\left({WF}_{i}\right)\right),℮\left(CPU\left({RP}_{i}\right)\right)*℮(TET\left(Dyna\;Q+\right))}{P(℮\left(length\left({WF}_{i}\right)\right)+℮(CPU\left({RP}_{i}\right)))},\;T\right)$$

$$\mathrm{Dyna}\;Q+:\;℮\left(\frac{WFET\left(Dyna\;Q+\right)}{A\Pi },\;T\right)≅low$$

$$E1=℮\left(\frac{WFET\left(E1\right)}{A\Pi },\;T\right)≅High$$

$$E2=℮\left(\frac{WFET\left(E2\right)}{A\Pi },\;T\right)≅Medium$$

$$E3=℮\left(\frac{WFET\left(E3\right)}{A\Pi },T\right)≅High$$

**PO2: Makespan Time** (MST(Dyna Q+)): The expected makespan time is influenced by the expected value of the maximum execution time of the resource pool.

$$℮(MST\left(Dyna\;Q+\right))=℮(Max(TET(Dyna\;Q+({RP}_{i})))$$

$$℮\left(\frac{MST\left(Dyna\;Q+\right)}{℮(Max(TET(Dyna\;Q+({RP}_{i})))\;},\;T\right)=\sum_{c\in C}c\int_{\alpha }^{\infty }\frac{\sum_{\alpha \epsilon \phi }℮\left(TET\left(Dyna\;Q+\right)\right)(a)}{\left|℮(Max(TET(Dyna\;Q+({RP}_{i}))\right|}$$

$$℮\left(\frac{MST\left(Dyna\;Q+\right)}{℮((Max(TET(Dyna\;Q+({RP}_{i})))\;},\;T\right)=℮\left(\frac{1*℮(Max(TET(Dyna\;Q+({RP}_{i})))}{P(℮((Max(TET(Dyna\;Q+({RP}_{i})))},\;T\right)$$

$$\mathrm{Dyna}\;Q+:\;℮\left(\frac{MST\left(Dyna\;Q+\right)}{A\Pi },\;T\right)≅low$$

$$E1=℮\left(\frac{MST\left(E1\right)}{A\Pi },\;T\right)≅Medium$$

$$E2=℮\left(\frac{MST\left(E2\right)}{A\Pi },\;T\right)≅High$$

$$E3=℮\left(\frac{MST\left(E3\right)}{A\Pi },\;T\right)≅Medium$$

**PO3: Operation Cost** (OC(Dyna Q+)): The expected value of the operation cost is influenced by the expected value of the cost incurred to process the requests by the resource pool.

$$℮\left(\frac{OC\left(Dyna\;Q+\right)}{℮(\sum_{k=1}^{3}\sum_{i=1}^{m}\left({C_{k}*D}_{i}\right))\;},\;T\right)=\int_{\alpha }^{\infty }\frac{\sum_{\alpha \epsilon \phi }℮(OC(Dyna\;Q+))}{\left|℮\left(length\left({WF}_{i}\right)\right),℮(CPU\left({RP}_{i}\right))\right|}$$

where, $D_{i}=TET(Dyna\;Q+({RP}_{i}))$.

$$℮\left(\frac{OC\left(Dyna\;Q+\right)}{℮(\sum_{k=1}^{3}\sum_{i=1}^{m}\left({C_{k}*D}_{i}\right))\;},\;T\right)=\sum_{c\in C}c\int_{\alpha }^{\infty }\frac{\sum_{\alpha \epsilon \phi }℮(OC\left(Dyna\;Q+\right))(a)}{\left|℮(Max(OC\left(Dyna\;Q+\right))\right|}$$

$$℮\left(\frac{OC\left(Dyna\;Q+\right)}{℮(\sum_{k=1}^{3}\sum_{i=1}^{m}\left({C_{k}*D}_{i}\right))\;},\;T\right)=℮\left(\frac{1*℮(Max(OC(Dyna\;Q+({RP}_{i})))}{P(℮\left(Max\left(\sum_{k=1}^{3}\sum_{i=1}^{m}\left({C_{k}*D}_{i}\right)\right)\right)},\;T\right)$$

$$\mathrm{Dyna}\;Q+:\;℮\left(\frac{OC\left(Dyna\;Q+\right)}{A\Pi },\;T\right)≅low$$

$$E1=℮\left(\frac{OC\left(E1\right)}{A\Pi },\;T\right)≅High$$

$$E2=℮\left(\frac{OC\left(E2\right)}{A\Pi },\;T\right)≅High$$

$$E2=℮\left(\frac{OC\left(E3\right)}{A\Pi },\;T\right)≅Medium$$

**PO4: Resource utilization rate** (RU(Dyna Q+)): The expected value of the resource utilization rate is influenced by the expected value of the memory load on the resource pool, $e(LM\left({RP}_{i}\right))$, and the CPU load on the resource pool, $e(LC\left({RP}_{i}\right))$.

$$℮\left(\frac{RU\left(Dyna\;Q+\right)}{\sum_{i=1}^{m}℮\left(LM\left({RP}_{i}\right)\right),℮(LC\left({RP}_{i}\right))\;},\;T\right)=\int_{\alpha }^{\infty }\frac{\sum_{\alpha \epsilon \phi }℮(RU(Dyna\;Q+))}{\left|\sum_{i=1}^{m}℮\left(LM\left({RP}_{i}\right)\right)+℮(LC\left({RP}_{i}\right))\right|}$$

$$℮\left(\frac{RU\left(Dyna\;Q+\right)}{\sum_{i=1}^{m}℮\left(LM\left({RP}_{i}\right)\right),℮(LC\left({RP}_{i}\right))},\;T\right)=\sum_{c\in C}c\;\int_{\alpha }^{\infty }\frac{\sum_{\alpha \epsilon \phi }℮\left(RU\left(Dyna\;Q+\right)\right)(a)}{\left|\sum_{i=1}^{m}℮\left(LM\left({RP}_{i}\right)\right)\_℮(LC\left({RP}_{i}\right))\right|}$$

$$℮\left(\frac{RU\left(Dyna\;Q+\right)}{\sum_{i=1}^{m}℮\left(LM\left({RP}_{i}\right)\right),℮(LC\left({RP}_{i}\right))\;},\;T\right)=℮\left(\frac{1*\sum_{i=1}^{m}℮\left(LM\left({RP}_{i}\right)\right)+℮(LC\left({RP}_{i}\right))*℮(RU\left(Dyna\;Q+\right))}{P(\sum_{i=1}^{m}℮\left(LM\left(RP\right)\right)+℮(LC\left({RP}_{i}\right))\;)},\;T\right)$$

$$\mathrm{Dyna}\;Q+:\;℮\left(\frac{RU\left(Dyna\;Q+\right)}{A\Pi },\;T\right)≅High\;E1=℮\left(\frac{RU\left(E1\right)}{A\Pi },\;T\right)≅High$$

$$E2=℮\left(\frac{RU\left(E2\right)}{A\Pi },\;T\right)≅Low$$

$$E3=℮\left(\frac{RU\left(E3\right)}{A\Pi },\;T\right)≅Medium$$

## 6. Results and Discussion

For the simulation of the proposed Dyna Q+ task scheduler, the CloudSim 3.3 simulator was used, which is one of the most widely used simulation tools for cloud computing environments [20]. CloudSim extends its support to simulate a wide range of virtual resources and allows for the experimentation of virtualized cloud data. Details on the simulation parameter setup are as follows: host (number of host = 30, MIPS = 188,770, bandwidth = 20 GB/s, storage = 3 TB, RAM = 16 GB, VM monitor = Xen), data center (number of data centers = 1, virtual machine scheduler = time shared, memory cost = 0.1–1.0, storage cost = 0.1–1.0, virtual machine monitor = Xen), client workflow (length of the workflow = 1 K–900 K, number of workflow = 300–1000), and virtual machine (number of virtual machine = 10–100, virtual machine speed = 4500–100,000 MIPS, memory = 1–4 GB, bandwidth = 2000–10,000, memory cost = 0.1–1.0, storage cost = 0.1 to 1.0, cloudlet scheduler = time shared, virtual machine monitor = Xen).

The efficiency of the proposed Dyna Q+ task scheduler is tested over three benchmark datasets: the random dataset, GOCJ dataset, and synthetic dataset. Comparisons of the proposed Dyna Q+ task scheduler against three of the existing task schedulers, E1 [15], E2 [18], and E3 [19], are conducted using performance metrics like task execution time, makespan time, operation cost, and resource utilization rate. The random dataset is composed of 1000 varieties of randomly generated workflows. This offers entirely random data for testing purposes. The dataset is generated using a built-in function of Python. It is composed of two columns: the index and value. The index represents the row ID and the value represents a randomly generated value. The GOCJ dataset is a realistic dataset generated using the bootstrapped Monte Carlo method. It comprises several files, and each file is composed of tasks in terms of millions of instructions. The tasks are derived from the workload behavior exhibited by Google cluster traces. The synthetic dataset is composed of random numbers that are generated using the Monte Carlo method for simulation. Typically, the client repeatedly requests the same kind of file, and the size of the file keeps varying in every test. This dataset allows the server to perform at its highest capacity since the requested file is stored in the server’s main memory.

### 6.1. Experiment 1: Random Dataset

The random dataset composed of four different workflow sizes, which are small (3 K–10 K), medium (20 K–40 K MI), large (50 K to 60 K MI), and extra-large (70 K to 79 K MI), is considered for evaluation purposes.

#### 6.1.1. Workflow Execution Time (WFET(Dyna Q+))

**Figure 2:**
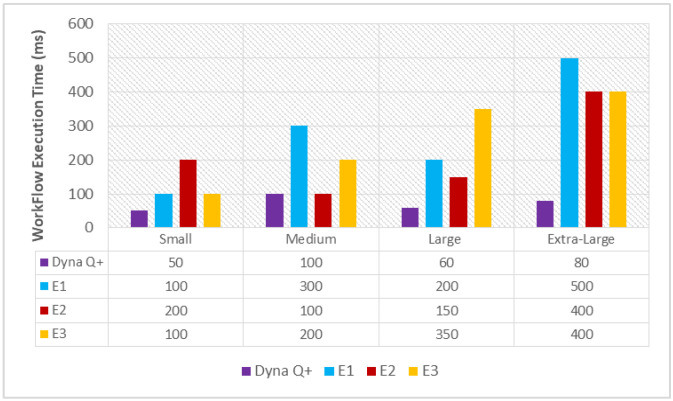
Different types of client workflows versus workflow execution time (ms).

A graph of different types of client workflows (small, medium, large, and extra-large) versus the WFET (ms) is shown in Figure 2. It is observed from the graph that the WFET of Dyna Q+ is consistently shorter for the entire variety of client workflows as the Dyna Q+ agent continuously keeps updating the rewards with respect to the time taken to formulate policies with the maximum number of rewards. On the other hand, the WFET of E1 becomes very long as the size of the Q-table grows exponentially in a large state space. Even the WFET of E2 and E3 is moderate due to the low precision of computation and the random selection of reference points for mimicking the foraging behavior of swarms.

#### 6.1.2. Makespan Time (MST(Dyna Q+))

**Figure 3:**
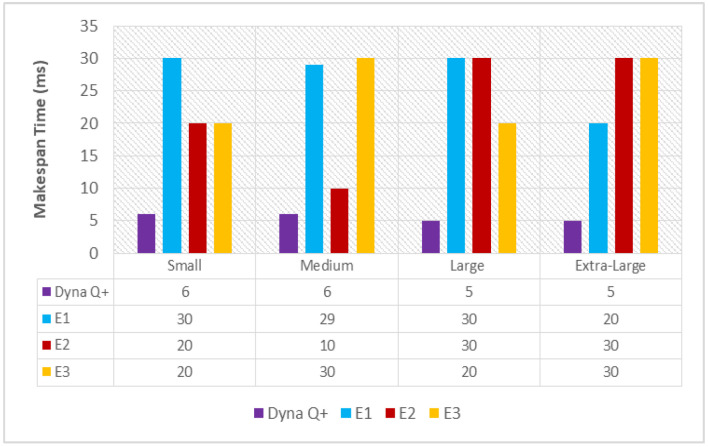
Different types of client workflows versus makespan time (ms).

A graph of different types of client workflows versus makespan time (ms) is shown in Figure 3. The MST of Dyna Q+ is very short for the entire variety of client workflows as the Q+ learning agent effectively remembers all the visited states through the exploration bonus. On the other hand, the MST of E1 and E2 is very long due to the improper balance between exploration and exploitation and the slow convergence speed. The MST of E3 is above a moderate length due to the opposition-based learning policy and poor tuning of the optimization parameters.

#### 6.1.3. Operation Cost (OC(Dyna Q+))

**Figure 4:**
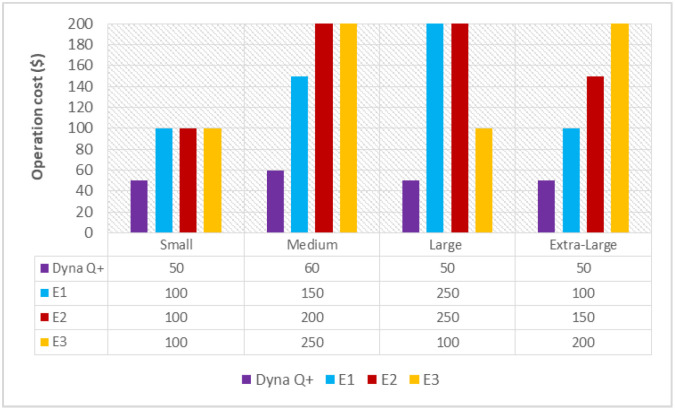
Different types of client workflows versus operation cost (ms).

A graph of different types of client workflows versus operation cost (ms) is shown in Figure 4. It is observed from the graph that the OC of dyna Q+ is lower for the entire variety of client workflows as it steadily increases the action value through repeated visits to unvisited areas. On the other hand, the OC of E1 is more than moderate as a large number of computational resources need to be stored and updated in the Q-table. The OCs of E2 and E3 are very high as they are highly sensitive to the choice of hyperparameters, and the agent’s action cannot be predicted from the swarm function.

#### 6.1.4. Resource Utilization Rate (RU(Dyna Q+))

**Figure 5:**
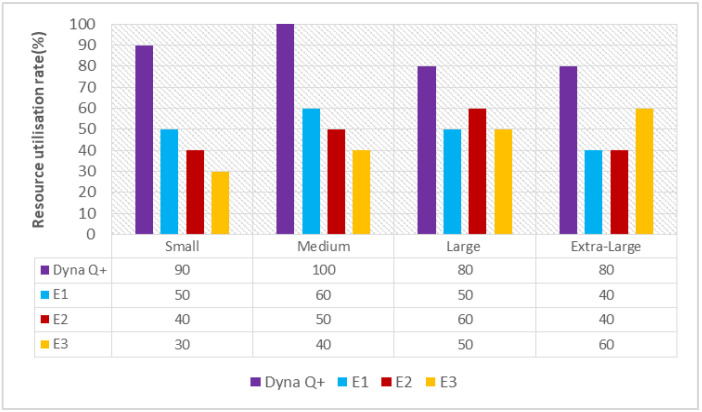
Different types of client workflows versus resource utilization rate.

A graph of different types of client workflows versus resource utilization rate is shown in Figure 5. The maximum amount of resources are utilized by the Dyna Q+ scheduler for the entire variety of client workflows, as obtaining a bonus for exploration leads to the Q+ agent having a faster learning rate. On the other hand, the RU rates of E1 and E2 are moderate due to their poor accuracy and the fact that it is easy for them to get trapped in moderate solutions. The RU of E3 is poor as it converges to a suboptimal solution even after training for many iterations.

### 6.2. Experiment 2: GOCJ Dataset

The GOCJ consists of a dataset that ranges from 20 K to 1000 K millions of instructions. It is composed of five different kinds of workflows, which are small-size workflows (15 K to 55 K millions of instructions), medium-size workflows (59 K to 99 K million instructions), large-size workflows (101 K to 135 K million instructions), extra-large-size workflows (150 K to 337 K million instructions), and huge-size workflows (525 K to 900 K MI).

#### 6.2.1. Workflow Execution Time (WFET(Dyna Q+))

**Figure 6:**
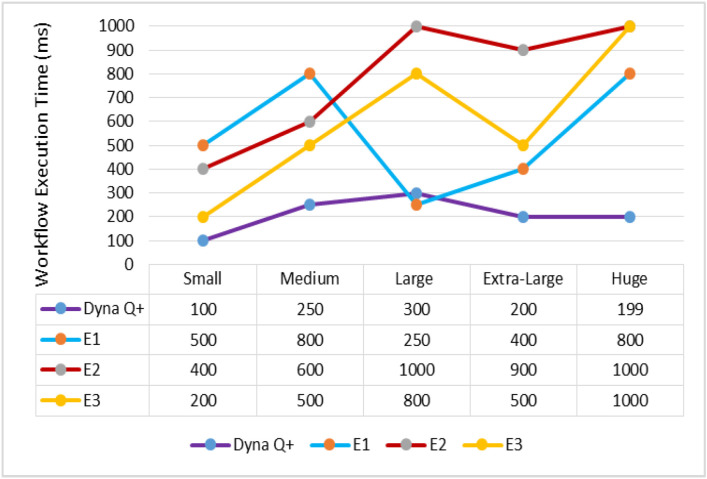
Different types of client workflows versus workflow execution time.

A graph of different types of client workflows (small, medium, large, extra-large, and huge) versus WFET (ms) is shown in Figure 6. The WFET of Dyna Q+ is very short for the entire variety of workflows, as it easily balances between exploration and exploitation over the large state space. The WFETs of E1 and E3 are moderate due to the self-updating of the Q-table and due to them mimicking chain foraging behavior. The WFET of E2 is very high for the entire variety of client workflows due to its restricted global search capability.

#### 6.2.2. Makespan Time (MST(Dyna Q+))

**Figure 7:**
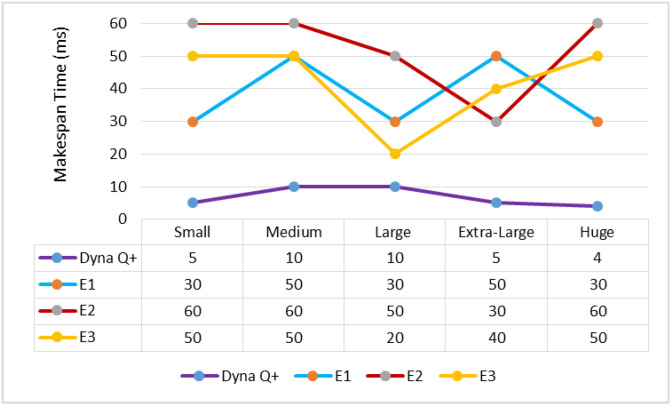
Different types of client workflows versus makespan time.

A graph of different types of client workflows versus makespan time (ms) is shown in Figure 7. The MST of Dyna Q+ is consistently shorter for the entire variety of client workflows, as it has an adaptable architecture that makes it suitable for dynamic environments. The MSTs of E1 and E3 are high due to the fine-tuning towards the optimal solution not being good. On the other hand, the MST of E2 is very long due to its poor global search capability.

#### 6.2.3. Operation Cost (OC(Dyna Q+))

**Figure 8:**
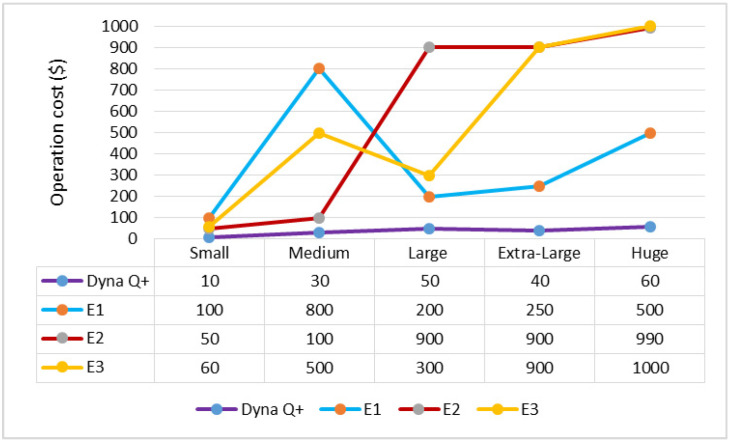
Different types of client workflows versus operation cost.

A graph of different types of client workflows versus operation cost (USD) is shown in Figure 8. The OC of Dyna Q+ is lower as it successfully operates in dynamic environments through action-based learning. The OC of E1 is moderate as the heterogeneous workflows are handled properly. The OCs of E2 and E3 are very high due to their poor local and global search capability to find a promising solution.

#### 6.2.4. Resource Utilization Rate (RU(Dyna Q+))

**Figure 9:**
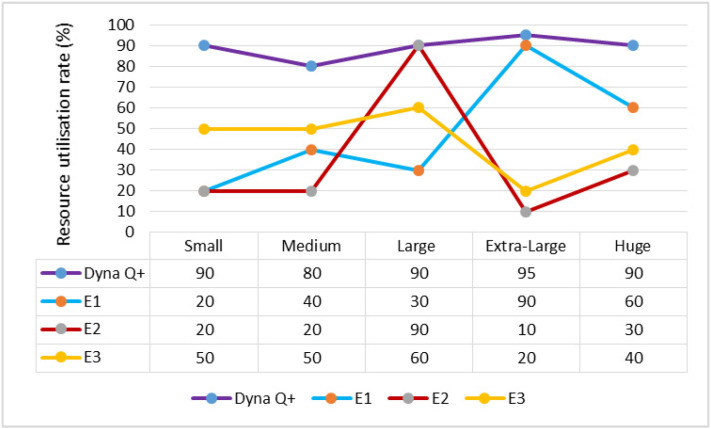
Different types of client workflows versus resource utilization rate.

A graph of different types of client workflows versus resource utilization rate is shown in Figure 9. It can be seen that the RU rate of Dyna Q+ is very high for the entire variety of client workflows because of the proper balance between the exploration and exploitation processes of the Q+ agent. The RU rates of E1 and E2 are lower as they easily become trapped in local optima. The RU rate of E3 is moderate as it takes the maximum amount of time for the search process to become saturated.

### 6.3. Experiment 3: Synthetic Dataset

The synthetic dataset is composed of five different varieties of workflows, which include tiny-size workflows (1 K to 250 K MI), small-size workflows (800 to 1200 MI), medium-size workflows (1800 to 2500 MI), large-size workflows (7 K to 10 K MI), and extra-large-size workflows (30 K to 45 K MI).

#### 6.3.1. Workflow Execution Time (TET(Dyna Q+))

**Figure 10:**
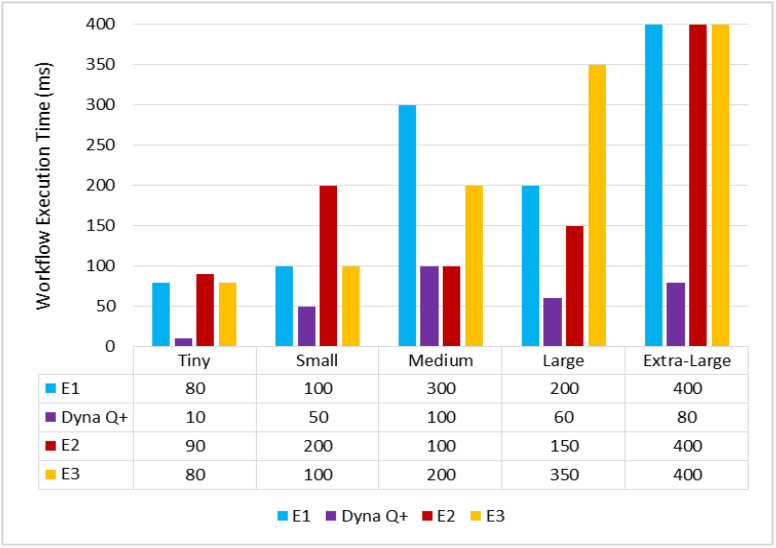
Different types of client workflows versus task execution time (ms).

A graph of different types of client workflows versus workflow execution time is shown in Figure 10. The WFET of Dyna Q+ is very short for the entire variety of workflows due to its convergence toward a promising solution through reward exploration. The WFETs of E2 and E3 are moderate due to their poor exploitation of the search space and their less adaptive control parameter strategies. On the other hand, the WFET of E1 is very high as chain foraging leads to the local optimum solution.

#### 6.3.2. Makespan Time (MST(Dyna Q+))

**Figure 11:**
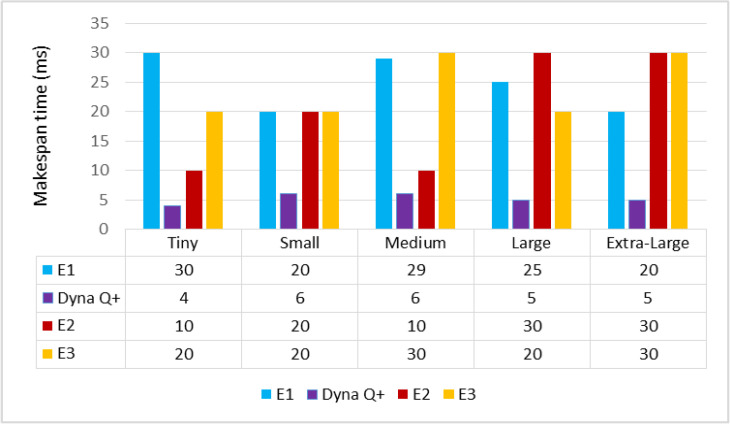
Different types of client workflows versus makespan time (ms).

A graph of different types of workflows versus makespan time is shown in Figure 11. It can be observed from the graph that the MST of Dyna Q+ is short as it extends more support for the exploration of large state spaces by gathering more cumulative rewards. On the other hand, the MST of E2 is moderate due to its inconsistent convergence speed when dealing with complex optimization problems. The MSTs of E1 and E3 are very long as the accuracy of the exploitation of large state spaces in later iterations of training is very low.

#### 6.3.3. Operation Cost (OC(Dyna Q+))

**Figure 12:**
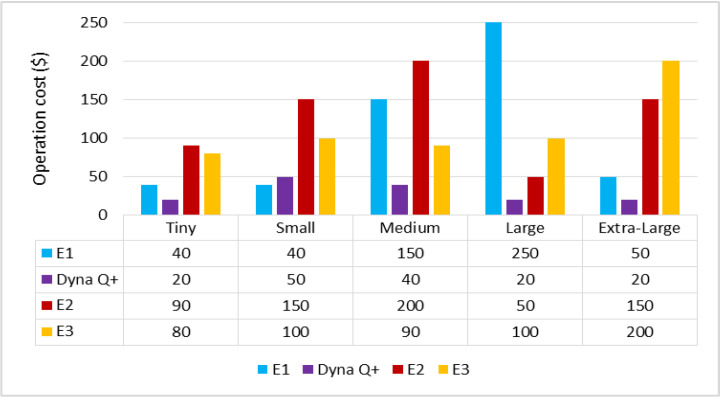
Different types of client workflows versus operation cost (USD).

The graph for different types of client workflows versus operation cost is shown in Figure 12. The OC of Dyna Q+ is low due to the exploration bonus. On the other hand, the OC of E1 is very high due to the pseudo-intermediate rewards present during task mapping. The OCs of E2 and E3 are moderate.

#### 6.3.4. Resource Utilization Rate (RU(Dyna Q+))

**Figure 13:**
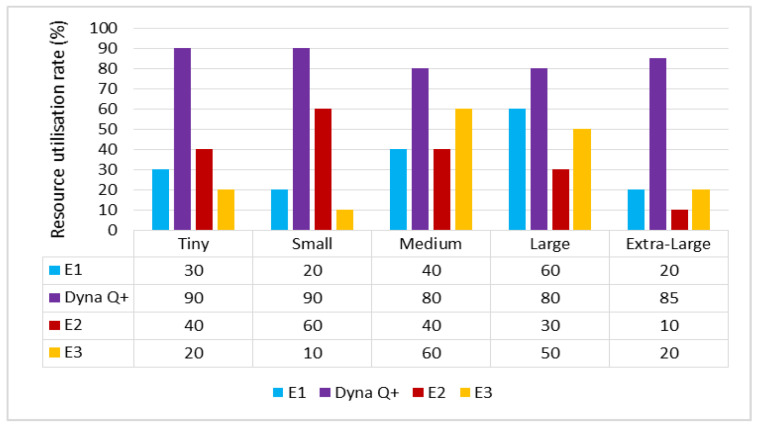
Different types of client workflows versus resource utilization rate.

A graph of the different types of client workflows versus resource utilization rate is shown in Figure 13. The RU rate of Dyna Q+ is very high due to its uniform random sampling of search spaces. The RU rate of E1 is low. The RU rates of E2 and E3 are very high due to them becoming trapped in local optima and their slower exploration of state spaces, owing to the diversity in the optimal solution.

## 7. Conclusions

This paper proposes a novel IVPFS-based Dyna Q+ task scheduler for cloud computing systems. The parameter uncertainty among the workflows and resource pools are handled via the application of the IVPFS mathematical framework. The proposed Dyna Q+ task scheduler is made uncertainty-proof and exhibits a high-adaptability feature for the changing dynamics of cloud systems by gathering exploration bonus rewards. The performance of the task scheduler is found to be good in terms of the following parameters: workflow execution time, makespan time, accuracy, and resource utilization rate. Its performance is further validated using expected value analysis, and the results are found to be satisfactory. The limitation of the proposed work is that it uses heterogeneous real-time dynamic cloud scenarios for testing. Our future work will concentrate on comparative analytical modeling of the scheduler by considering dynamic cloud scenarios.

## Data Availability

The simulation of the proposed “IVPFN-Dyna Q+ task scheduler in cloud environment” utilized three workloads: a random dataset, GOCJ dataset, and synthetic dataset selected from [16,17].
